# Composite Nanogels Based on Zeolite-Poly(ethylene glycol) Diacrylate for Controlled Drug Delivery

**DOI:** 10.3390/nano10020195

**Published:** 2020-01-22

**Authors:** Catalina Paula Spatarelu, Anita-Laura (Radu) Chiriac, Bogdan Cursaru, Tanta-Verona Iordache, Ana-Mihaela Gavrila, Crina-Thea Cojocaru, Razvan-Edward Botez, Bogdan Trica, Andrei Sarbu, Mircea Teodorescu, Vlad Tofan, Francois-Xavier Perrin, Anamaria Zaharia

**Affiliations:** 1 Advanced Polymer Materials and Polymer Recycling Group, National Institute for Research and Development in Chemistry and Petrochemistry-ICECHIM, 202 Spl. Independentei, 6th District, P.O. Box 35/174, 060021 Bucharest, Romania; spatarelucatalina@gmail.com (C.P.S.); bcursaru17@gmail.com (B.C.); iordachev.icechim@gmail.com (T.-V.I.); anamihaela.florea@gmail.com (A.-M.G.); crina.thea@gmail.com (C.-T.C.); razvan.botez@yahoo.com (R.-E.B.); trica.bogdan@gmail.com (B.T.); andr.sarbu@gmail.com (A.S.); 2 Department of Bioresources and Polymer Science, Faculty of Applied Chemistry and Materials Science, University “Politehnica” of Bucharest, 1–7 Gh. Polizu Street, 1st District, 011061 Bucharest, Romania; mircea.teodorescu@upb.ro; 3 Cantacuzino National Institute of Research-Development for Microbiology and Immunology (CNIR), 103 Spl. Independenţei, 5th District, 011061 Bucharest, Romania; tofan.vlad@gmail.com; 4 MAPIEM Laboratory, University of Toulon, EA 4323, 83130 La Garde, France

**Keywords:** composite nanogels, zeolite, poly(ethylene glycol) diacrylate, mini-emulsion polymerization, 5-FU, controlled release

## Abstract

This study presents the design of novel composites nanogels, based on poly(ethylene glycol) diacrylate and natural zeolite particles, that are able to act as materials with controlled drug delivery properties. Natural zeolite–nanogels composite, with varying zeolite contents, were obtained by an inverse mini-emulsion technique and loaded with 5-fluorouracil, a widely used chemotherapeutic drug. Herein, the possibility of adjusting final properties by means of modifying the preparation conditions was investigated. The prepared composite nanogels are characterized by dynamic light scattering (DLS), transmission electron microscopy (TEM), Fourier transform infrared spectroscopy (FTIR), and thermogravimetric analysis (TGA). In light of this tunable drug-loading capability, swelling behaviour, and cytotoxicity, these composite nanogels could be highly attractive as drug reservoirs.

## 1. Introduction

The application of nanotechnology in the pharmaceutical field is focused on developing proper therapeutic agents, composed mainly of existing medicines and their carriers. The need for designing such systems as an alternative cancer therapy arises from the drawbacks of administering the native medicine directly. These issues are related to the low solubility of the drugs, toxicity to healthy tissue, inefficient penetration of biological barriers, or in vivo instability, all of which can drastically limit the successful use of the drugs [1,2]. Nanocarriers are nanocolloids able to transport biologically active compounds, including low-molecular-weight compounds or biomacromolecules, such as genes or proteins [3,4]. They can consist of polymer-conjugated systems, polymeric nanoparticles (NPs), lipid-based carriers (liposomes, micelles), dendrimers, carbon nanotubes, or inorganic NPs (gold, zeolites, silica, etc.). The design of a delivery system should take into account the envisioned application, the nature of biological compound, material safety, and the administration route [2,3]. The system, ideally, will not only address the shortcomings of the native drug, but also convey several advantages such as a high loading capacity, protection against in vivo degradation, higher cytostatic doses, and their subsequent controlled and slow release [5,6,7,8].

Recently, so-called nanogels (NGs), polymeric NPs based on hydrogels, represent a convergence of NPs and hydrogels, with both systems suitable for drug delivery [9]. The combination of the hydrophilicity, versatility, biocompatibility, and high water retention, together with the prolonged circulation times and enhanced permeation and retention at tumour sites due to the size, make NGs a top candidate when designing and developing passively targeted therapy [10]. When employing NGs as carriers, biologically active compounds, either lyophilic or hydrophilic low-molecular-weight molecules, or biomacromolecules (DNA, RNA, proteins, etc.) [11] are encapsulated in the three-dimensional network. The loading can be done by physical entrapment or through noncovalent interactions. This method is also suitable for poorly soluble anticancer medicines as it confers biostability, ensuring they are internalized by the targeted cells. In this context, it becomes worth mentioning the paper of Li et al., which proposes the use of Pluronic F127 to form paclitaxel- and 10-hydroxycamptothecin-loaded nanogels [12] for the passive targeting of cancer cells. The development of composite NGs based on nanomaterials [13], such as clays, carbon nanotubes, and magnetic NPs, attracted real interest in the nanogels field. Natural or synthetic zeolites are microporous crystalline solids with well-defined structures, showing biocompatibility, nontoxicity, large surface areas, and high pore volumes. All these properties render natural zeolites suitable for the biomedical field as controlled release systems [13,14,15] and haemostatic agents [16]. Sotoudeh et al. [17] have obtained nanocomposite hydrogels based on polyethylene glycol, polyacrylic acid, and polyacrylamide with zeolite NPs, which were tested in terms of the controlled release of drugs. Also, Zhang’s team [18] synthesized polymeric composites by inverse emulsion polymerization, using chitosan, gelatine, and alginate with zeolite. The new structure of microgels exhibited haemostatic abilities as well as the controlled release of antibiotics.

The novelty of the present study consists of the obtaining of cross-linked poly(ethylene glycol) diacrylate NGs containing natural zeolite NPs loaded with bioactive substances. The preparation of composite NGs based on poly(ethylene glycol) diacrylate and zeolite used as controlled release systems is a new topic in this field [18]. Natural zeolites exhibit various biological activities, being successfully used as compounds that activate a drug (anticancer therapy, vaccine, etc.) [19].

The present study deals with the synthesis and characterization of cross-linked poly(ethylene glycol) diacrylate nanogels (NGs PEGDA) obtained by inverse mini-emulsion and containing natural zeolites (NZ), loaded with 5-fluorouracil (5FU), a bioactive substance (SBA) used in cancer treatment. The incorporation of the natural nanozeolite type B used in this study allowed for a much slower and more controlled release of the embedded active substances and improved the viscoelastic properties of the final composite hydrogels [20,21,22]. The inverse mini-emulsion polymerization system involves the formulation of a stable mixture, composed of droplets of a polymer aqueous solution suspended by a mixture of co-surfactants in a continuous organic medium [23]. Herein, we investigate the influence of several synthesis parameters on the final properties of the obtained NPs. The prepared NG composites are characterized by dynamic light scattering (DLS), transmission electron microscopy(TEM), Fourrier-Transforminfrared spectroscopy (FTIR), and thermogravimetric analysis (TGA). The composites were analysed in terms of their swelling behaviour, in vitro drug release profiles, and cytotoxicity activity to assess their potential as drug delivery systems.

## 2. Experimental

### 2.1. Materials 

In order to obtain the desired composite NGs, the following reagents were used: poly(ethylene glycol) diacrylate PEGDA (MW=700 g/mol, Sigma-Aldrich, St. Louis, MO, USA), PEGDA (M_W_=2000 g/mol, laboratory synthesized), tetramethylenethylenediamine (TMEDA, Merck, Darmstadt, Germany, 99%), ammonium persulfate (APS, Peking Chemical Works, Beijing Chemical Works, Beijing, China, 98%), sorbitan monooleate (SPAN 80, Sigma-Aldrich, St. Louis, MO, USA), polyethylene glycol sorbitan monooleate (TWEEN 80, Fluka, Fluka Chemie GmbH, Buchs, Switzerland), NaCl (Sigma-Aldrich, St. Louis, MO, USA, >99%), 5-fluorouracil (5-FU, Sigma-Aldrich, St. Louis, MO, USA, >99%), dimethyl sulfoxide (DMSO, Fisher Chemical, Fisher Scientific, Pittsburgh, PA, USA), phosphate-buffered saline (PBS, Roti^®^-CELL),and acetone were used as received. The natural zeolite type B was provided by the National Institute of Rare and Non-Ferrous Metals, Bucharest. 

### 2.2. Preparation of 5-FU-Loaded Nano-Zeolite (NZ) Particles

The natural zeolite with the lowest particle dimension among the provided samples was further grinded with the help of a planetary ball mill PM100, 230 V, 50/60 HZ, until reaching submicronic sizes. The milling was done under dry conditions, at 300 rpm, in five stages of 1 min each, with intermediary breaks of 30 s, using 10 stainless steel balls with a 5 mm diameter. Before loading with 5-FU, the NZ particles were dried overnight at 120 °C to remove the physiosorbed water. The 5-FU-loaded NZ particles were prepared by immersing NZ particles in a DMSO solution of 5-FU (50 mg/mL), ultrasonicated at room temperature for 2 h followed by magnetic stirring at 300 rpm for 22 h. After loading, the mixture was centrifuged and the supernatant removed and replaced with fresh DMSO (2 × 30 mL). This process was required to remove the drug adsorbed on the surface. The loaded NZ particles were lyophilized at −110 °C for 48 h to remove the solvent [24]. 

### 2.3. Synthesis and Purification of Composite NGs by Inverse Mini-Emulsion Polymerization

The composite NGs were obtained by inverse mini-emulsion polymerization (oil–water), which involves a stable mixture of water-soluble polymer micelles by means of oil-soluble surfactants and co-surfactants in a continuous organic medium [25,26]. SPAN 80 and TWEEN 80 were mixed thoroughly in a 7:1 ratio (*w*/*w*) with cyclohexane in a 50-mL one-neck round-bottomed flask. The ratio of aqueous/organic media was 1:5.35 (*w*/*w*), and the total concentration (wt%) in the organic phase of co-surfactant mixture used was of 5.72%. The SPAN 80/TWEEN 80 combination yielded a hydrophilic–lipophilic balance (HLB) value of 5.6. The mixture was homogenized and purged with N_2_ for 5 min. Meanwhile, the aqueous phase was prepared by dissolving the corresponding quantity of macromer (PEGDA_2000_, PEGDA_700_ or ablend of the two containing 25% PEGDA_2000_) and TMEDA (~0.5 wt% with respect to the diacrylate groups in the macromer) in water and adding the 5-FU-loaded NZ. Additionally, NaCl was added in a 0.5% molar proportion to the macromer(s) to avoid Oswald ripening of the mini-emulsion. To get a proper dispersion of the inorganic phase in water, the mixture was ultrasonicated and sparged with N_2_ to remove any dissolved O_2_. The two phases were mixed under quick stirring (700 rpm), and APS (10% solution) was added, to initiate the polymerization reaction. The total reaction time was 42 h, after which the mixture was centrifuged, the supernatant removed, and the gel phase washed with cyclohexane (2 × 30 mL), acetone (1 × 30 mL), and water (1 × 30 mL). The washing steps are meant to remove the continuous media, as well as the emulsifiers and any unreacted macromer. After purification, the composites were lyophilized for 48 h, yielding a powder. The conversion was computed as follows:

(1) $$conversion=\frac{m_{weighted}}{m_{theoretical}}\times 100$$

where $m_{weighted}$ is the weighted mass of the lyophilized composite and $m_{theoretical}$ is the sum of the individual components that make up the composite (monomers, zeolite, lipophobic agent where used), as weighed at the beginning of the polymerization reaction.

The composite NGs are designated as *x*NZ-PEGDA*_y_*, where *x* represents the zeolite content (wt%) and *y* represents the molecular weight of PEGDA used. The synthesis recipes of the composite NGs are presented in Table 1, along with control NGs without zeolite. For the FTIR, swelling trials and cell viability studies, unloaded NZ-NG composites were also prepared using the same recipe but with plain NZ. 

### 2.4. Swelling Degree Determination

The swelling degree (SD) of control NGs and corresponding composite NGs was determined by the immersion of previously weighed dried samples in a known PBS volume for seven days at 37 °C. At the end of the swelling period, the swollen samples were weighed in order to determine the swelling degree via Equation (2):

(2) $$\mathrm{SD},\;\%=\frac{m_{wet}-m_{dry}}{m_{dry}}\cdot 100$$

where *m_wet_* and *m_dry_* denote the weight of swollen NG and the weight of the dry NG, respectively.

### 2.5. Release Study of 5-FU from Composite NGs

The study was carried out by dialysis, using a dialysis membrane SpectraPor^®^ (cutoff = 6–8 kDa). In a dialysis bag for drug release, a known amount of sample was introduced to 2 mL PBS (pH = 7.4), which was in turn immersed in a fresh PBS bath and maintained at 37 °C for the duration of thestudy. At different time intervals, 2 mL of solution were collected from the PBS outside the membrane, and the solution was replenished with an equal amount of fresh PBS. The samples were analysed in triplicate to ensure the accuracy and reproducibility of the results. Before the measurement of the collected solutions, a calibration curve was drawn, in the linear concentration range of 5-FU by UV-VIS spectrometry, allowing for the correlation of absorbance and sample concentration. 

The concentration of the 5-FU released was determined according to Equation (3):

(3) $$C_{t\;cummulative}=C_{t}+\frac{\vartheta }{V}\sum_{0}^{t-1}C_{t}$$

where $C_{t\;cummulative}$ is the corrected concentration at time *t*, taking into account the volume changes, $C_{t}$ is the apparent concentration at time *t*, *ν* is the sampled volume, and *V* is the total bath volume. To assess the difference in the initial mass of hydrogels, the results are reported as a percentage of the loading of the composites. The following mathematical models were used to analyse the release profile and release mechanism [27,28]:

First-order model:

(4) $$conc_{t}=conc_{0}\exp\left(-k_{1}t\right)$$

Zero-order model:

(5) $$conc_{t}=conc+k_{0}\cdot t$$

Higuchi model:

(6) $$conc\;=\;K_{H}\cdot \sqrt{t}$$

Korsmeyer–Peppas model:

(7) $$\frac{M_{t}}{M_{\infty }}=k\cdot t^{n}$$

where *conc_t_*, *conc_o_* is the amount of drug released at time *t* and the amount of initial drug in the sample, ‘conc’ is the initial amount of drug in the solution, *k_1_* is the first-order rate constant, *k_o_* is the zero-order constant, *K_H_* is the Higuchi dissolution constant, *M_t_*/*M_∞_*, is the fraction of drug released at time *t*, *k* is a kinetic constant characteristic of the drug–polymer system, and ‘*n*’ is a release coefficient that characterizes the 5-FU transport. 

### 2.6. Cytotoxicity Study 

The cell line L929 was obtained from ECACC and was used to estimate the cytotoxicity of the nanocomposites. The confluent culture was passaged at a ratio of 1/8. The culture medium was Dulbecco’s Modified Eagle Medium (DMEM), supplemented with 10% foetal bovine serum (FBS), 100 μg/mL penicillin, 100 μg/mL streptomycin, and 4 mM L-glutamine. After 24 h, the cells were detached from the vessel surface by trypsinization and resuspended in culture medium. In order to determine the cellular concentration (number of cells/mL medium), the cells were coloured with Trypan Blue (dilution ½). For counting, a haemocytometer and an inverse optic microscope Eclipse TE2000 were used. In order to reach a concentration of 10,000 cells/mL for the seeding of each well, the cells were centrifuged and diluted in culture medium. A 96-well plate was seeded, with each sample being duplicated. After the homogenization of the suspension, 100 μL/well were added and the samples were incubated at 37 °C, under controlled humidity and 5% CO_2_ atmosphere, for 4h, to allow for adhesion. Samples of NGs were left in PBS to allow the elution of the constituents. After twodays the supernatant was filtered in a sterile manner using a 0.2-µm filter. Next, the culture medium was removed and the samples’ supernatant was added, starting at a 1/10 dilution ratio in the culture medium, and continuing with binary dilutions. The cells were incubated under the same conditions overnight. After the incubation, a 3-(4,5-dimethylthiazol-2-yl)-2,5-diphenyltetrazolium bromide(MTT) assay was performed to evaluate the cell viability. The cell viability percentage was determined according to Equation (8):

(8) $$Cell\;Viability,\;\%=\frac{A_{t}}{A_{c}}\cdot 100$$

where $A_{t}$ is the absorbance of cell treated with sample and $A_{c}$ is the absorbance of the untreated cells.

### 2.7. Characterization Techniques

The particle sizes of control and composite NGs, as well as the dispersed zeolite, were determined by dynamic light scattering (DLS) analysis using a Malvern Zetasizer Nano-ZS (Malvern Instruments, Worchestershire, UK) system equipped with a 4 mW He–Ne laser (633 nm). The zeolite was dispersed in cyclohexane (1 g in 1 mL) and introduced to an ultrasonic bath before analysing in order to prevent the particle agglomeration tendency. The particle size in the case of simple and composite nanogels was measured for the diluted emulsions (sample collected directly from the synthesis reaction and diluted with 1 mL cyclohexane) and for the supernatant (the gel phase washed with cyclohexane). All measurements were done as five replicates, and the result reported as the mean together with the standard deviation.

The X-ray diffraction (XRD) patterns were collected with a SmartLab diffractometer (Rigaku, Tokyo, Japan) equipped with Cu Kα radiation (wavelength k = 0.1541 nm). The scanned range was 2Θ = 2–40, with a scan speed of 1° min^−1^.

Transmission electron microscopy (TEM) pictures were taken using a Tecnai™ G2 F20 TWIN Cryo-TEM Field Electron and Ion Company (FEI) Corporate Headquarters, Hillsboro, OR, USA). Two protocols were used. The first one consisted of directly sampling the emulsion and placing it on a carbon-film-covered grid. The excess emulsifiers are removed by a 5 s immersion in acetone of the grid. The second one consisted of the redispersion of the purified composites in distilled water and placement of a sample on the same type of grid. Bright-field TEM (BFTEM) was carried out at an acceleration voltage of 200 kV, after confirming that the morphology is not affected by the large exposure. Bright field scanning transmission electron microscopy (BFSTEM) images were also obtained to provide more information on the studied materials. 

The Fourier transform infrared (FTIR) spectra of the samples were recorded on a Nicolet FTIR Model 500 (Thermo Fisher Scientific, Walthman, MA, USA) by acquiring 32 scans with 4 cm^−1^ resolution in the 4000–400 cm^−1^ region, on a KBr pellet. 

The thermogravimetric analyses (TGA) were performed on a Thermal Analysis SDT600 (TA Instruments, New Castle, DE, USA) instrument by heating 5–10 mg samples from 30 °C to 1000 °C at a heating rate of 10 °C/min under a nitrogen flow.

Diffuse reflectance UV (DR-UV) spectra of the solid samples were recorded on a UV-1800 Shimadzu^®^ spectrometer (Shimadzu Scientific Instruments Inc., Kyoto, Kyoto Prefecture, Japan) equipped with an integrating sphere. 5-FU samples were diluted with BaSO_4_. BaSO_4_ and untreated zeolite were used for the blank spectrum in the case of 5-FU or with 5FU-NZ powder, respectively. The ultraviolet-visible spectroscopy (UV-Vis) analysis for drug release experiments were performed on an EVOLUTION 260 BIO (Thermo Fisher Scientific, Walthman, MA, USA) device by measuring the absorbance of the solutions at λ = 265 nm for 5-FU. The amount of drug released was determined from the corresponding calibration curve, obtained using solutions of known concentrations. 

## 3. Results and Discussion

### 3.1. Characterization of the 5-Fluorouracil-Loaded Zeolite Nanoparticles

#### 3.1.1. DR-UV-Vis Measurements

The loading of 5-FU in the NZ particles was done by dissolving the drug in DMSO. According to literature studies [24], DMSO has a good solubility towards the drug, which encourages an increased loading of the porous matrix compared to other solvents (water, water–acetone, ethanol, etc.). 

**Figure 1:**
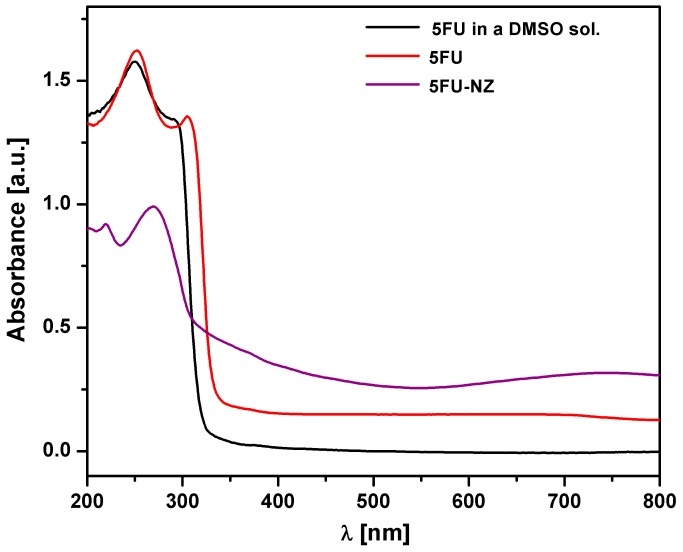
DR-UV-Vis spectra of 5-FU, 5-FU-loaded NZ (5-FU-NZ), and lyophilized 5-FU in a dimethyl sulfoxide (DMSO) solution.

The DR-UV spectra for 5-FU and for the 5-fluorouracil-loaded zeolite (5FU-NZ) are shown in Figure 1. The presence of 5-FU in 5FU-NZ is evidenced by the intense absorption band centred around 277 nm arising from the HOMO-LUMO π/π* transition [29]. The 5-FU peak in 5FU-NZ is ca. 25–27 nm red-shifted with respect to that of 5-FUdispersed in BaSO_4_(~252 nm). Knowing that the zeolite was loaded from a DMSO solution, the analysis of an equivalent amount of 5-FU dissolved in DMSO and lyophilized under the same conditions was necessary to confirm that the redshift is not due to interactions between the 5-FU and the residual solvent (DMSO). As seen in Figure 1, the DR-UV spectra of the two 5-FU samples are very similar. Therefore, the red shift that appears in the case of 5-FU-loaded NZs might be the result of interactions between the NZ particles and 5-FU. Potential interactions could consist of: (i) carbonyl or amino functionalities of 5-FU creating complexes with Al^3+^ from the aluminosilicate matrix; and (ii) NH groups or oxygen atoms (with a strong electronegative character) creating hydrogen bonds with H atoms from hydroxyl groups present in the zeolite (present as Si–OH).

#### 3.1.2. TGA Study

**Figure 2:**
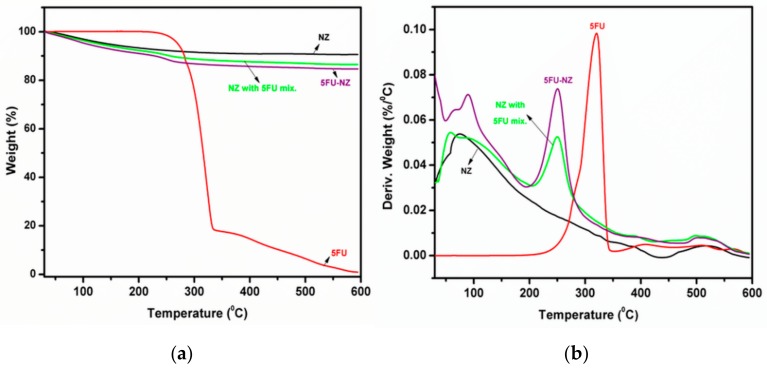
TGA (**a**) and DTG (**b**) curves of zeolite (NZ), 5FU, and 5FU-loaded zeolite particles (5FU-NZ).

The content of the loaded zeolite was estimated by means of thermogravimetry. The TGA data were used to calculate the 5-FU loading. Figure 2 shows the TGA and DTG curves of the pure drug molecule and zeolite before and after 5-FU loading. The mass loss for the loaded zeolite takes place in two main stages. The first one in the 50–200 °C range results from the removal of physisorbed water in the zeolite. The DTG signal in the range of 50–200 °C is very similar for empty and loaded zeolite, which indicates a very low amount of remnant DMSO solvent in the loaded zeolite. It also indicates that the loading treatment has not affected the hydration state of the zeolite. The next step takes place in the temperature range 200–600 °C and corresponds to the decomposition of the 5-FU molecule. It is noteworthy that the maximum DTG peak for 5-FU appears at a much lower temperature in the loaded zeolite compared to the degradation temperature of the free drug molecule. This is a further indication of interactions of the zeolite framework and the drug molecule [30]. The total amount of 5-FU in the zeolite is 7.4% relative to wet zeolite and 8.3% relative to dry zeolite. 

#### 3.1.3. XRD Patterns

**Figure 3:**
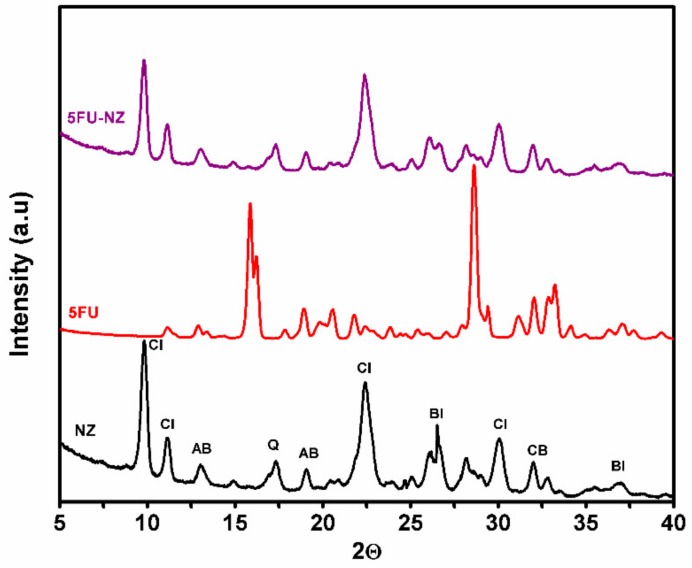
X-ray diffraction XRD patterns for zeolite (NZ), 5FU, and 5FU-loaded zeolite particles (5FU-NZ).

Figure 3 shows the XRD pattern of zeolite, 5-FU, and 5-FU-loaded zeolite. The XRD results indicate the presence of clinoptilolite (Cl) as a major crystalline phase, as well as peaks corresponding to the biotite (BI), quartz (Q), cristobalite (CB), and albite (AB) phases. The structural and textural properties for the natural zeolite indicated the following composition: Cl71–73%; AB12%; BI9–10%; Q2–3%, and CB2.7–3.1%. The absence of diffraction peaks at high angles (over 10–40 degrees) maybe due to a packing of weakly defined pores within the zeolite material [31]. The XRD patterns after 5-FU loading were similar to the XRD pattern of the parent zeolite, indicating that the frameworks did not undergo any significant structural change during the 5-FU loading procedure. Moreover, the absence of any peaks associated with 5-FU in the 5-FU-loaded sample suggests the absence of crystalline 5-FU. Considering that the drug’s presence was confirmed by previous tests, this result might signify that the drug is present within the pores rather than on the surface of the matrix, where it would have had the opportunity of crystallizing, hence being visible in the diffraction spectra.

### 3.2. Characterization of Control and Composite NGs

#### 3.2.1. DLS Investigation

In our initial attempts at synthesizing the composites, the polymerization reaction was observed to have poor yields (Table 2, see 3NZ-PEGDA_700_) and exhibited a phase separation of the particles at the end of the reaction. This can be explained by a phenomenon known as Ostwald ripening, which causes the macromer to leak out of the water droplets in a process of inverse emulsion, thereby lowering the quantity of macromer that gets to react [32]. In order to avoid this pitfall, as well as increase the yield and mini-emulsion stability, a strong lipophobic agent was added (NaCl) at 0.5% with respect to the macromer, to decrease the chemical potential of the dispersed media. According to the obtained results, outlined in Table 2, a drastic improvement in reaction yield (conversion) was observed, confirming that the addition of NaCl was beneficial. In order to avoid micellar nucleation, SPAN 80′s concentration was kept below its critical micellar concentration value (CMC of ∼3.2 g in 80 mL of cyclohexane or 5.15 wt%). 

**Figure 4:**
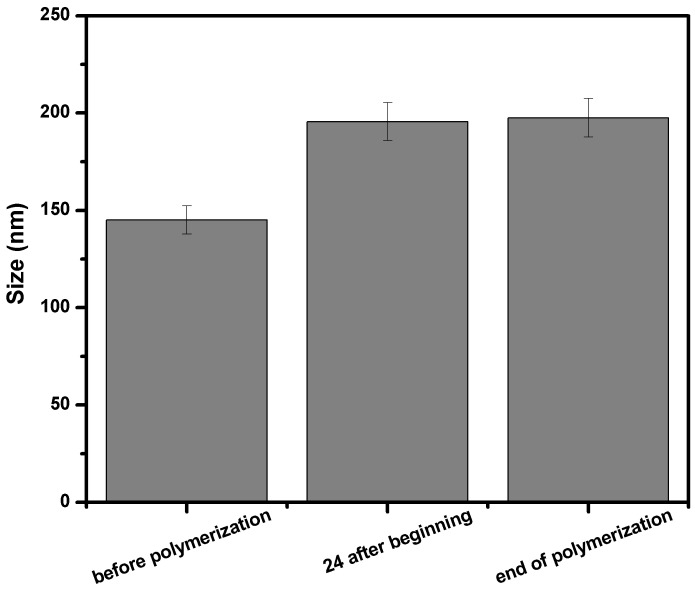
Mean particle size before, during, and after the polymerization process.

Moreover, in order to gain insight into the polymerization mechanism, and to confirm that the process corresponds to a mini-emulsion (also known in the literature as a nanoemulsion), mean size measurements were carried out for the PEGDA_700_ specimen before the start of the reaction, after 24 h of polymerization, and at the end of the reaction (42 h) (see Figure 4). Given the sizes were roughly similar, the measurement indicated that the reaction is characteristic of an inverse mini-emulsion system, in which each water droplet acts as a nanoreactor where the polymerization takes place in bulk [33].

Important parameters of mini-emulsion, such as the hydrophilic–lipophilic balance of the surfactant (HLB), the surfactant/aqueous and the solvent/aqueous medium ratio, the monomer concentration, the molecular weight of the PEGDA oligomer, and the blending ratio of the two different macromers (PEGDA_700_ and PEGDA_2000_) were previously studied for their effects upon the particle size, size distribution, and morphology of these advanced NGs [34]. Thus, the mixture between a monomer with short length (PEGDA_700_) and a monomer with longer chains (PEGDA_2000_) generated a network with a special architecture. However, the network was not perfect due to the higher concentration of acrylic end groups, which led to higher cross-linking degrees and network interferences. Furthermore, it was observed that the hydrophilicity of NGs was greater when PEGDA with a higher molecular weight was used. Hence, preparing NGs using two polymeric blocks with different hydrophilicity, mobility, and biocompatibility (greater for PEGDA_2000_) [35,36] constituted a mechanism for tuning the hydrophilicity of particles (a highly important property for potential drug delivery applications). In this context, DLS analysis revealed that the addition of a small amount of PEGDA_2000_ in the reaction mixture yields NG particles with small sizes and nearly unimodal distribution [34].

The composite NGs were studied for their size distribution as well as their polydispersity, following the influence of the varied parameters, i.e., incorporated zeolite percentage and type of macromer. The DLS data are presented in Table 3, where HLB is the hydrophilic–lipophilic balance and PDI is the polydispersity index. For the synthesis of composite NGs, the weight percentage of added zeolite varied (in the range 1–3%), together with the variation of macromer (PEGDA_2000_, PEGDA_700_, PEGDA_2000_-PEGDA_700_(1:3) blend). For the blend with 25 wt%PEGDA_2000_, in all the studied loadings, the experiments yielded the lowest value in terms of mean particle size. An increase in the weight percentage of zeolite led to an increase in the mean particle size. At the same time, the mini-emulsion polydispersity increased with the content of inorganic matrix. This could be due to the zeolite agglomeration tendency in relatively low amounts of water. 

The mechanism of NPs’ binding to biological matter is not yet fully understood. In general, toxic compounds are described by well-established sets of available methodologies. Nevertheless, the bioassays involving nanomaterials are still under development and have not yet been internationally validated. One of the main reasons is that there are several variables involved when working with NPs, including material size, shape, surface charge, agglomeration degree, and concentration. For instance, particles of the same material can show completely different behaviour due to slight differences in size. This makes the evaluation of NPs’ cytotoxic effect difficult and, thus, the reproducibility may vary in each assay. In other words, the polydispersity (PDI) of NPs should be very low in order to obtain comparable results in each batch. This is why the cytotoxicity results should also be linked to the PDI of NPs.

The fact that, for the composite NGs containing 3% NZ, the mean size was registered within the desired range (100–200 nm ± 10%) was a good indicator of the possibility of working with an increased loading of NZs. Given the targeted application, an increased percentage of zeolite is translated into an increased quantity of biologically active drug in the final composite.

#### 3.2.2. TEM Images

**Figure 5:**
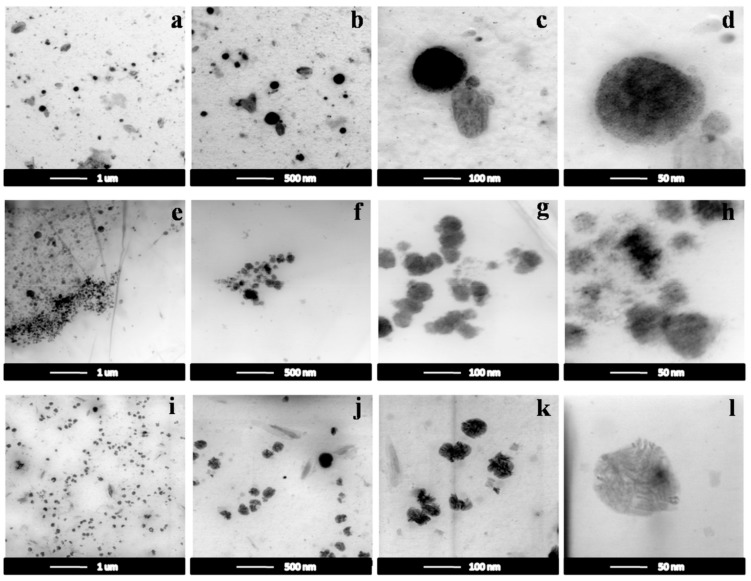
Images for the emulsion of simple NGs: (**a**) 1 μm; (**b**) 500 nm; (**c**) 100 nm; (**d**) 50 nm; and NG nanocomposites with a 2% NZ ((**e**) 1 μm; (**f**) 500 nm; (**g**) 200 nm; (**h**) 50 nm) and 3% NZ ((**i**) 1 μm; (**j**) 500 nm; (**k**) 200 nm; (**l**) 50 nm) NZ content.

The control and composite NGs were characterized in terms of morphology (TEM), in order to reveal the morphology of the composite NGs. Figure 5 shows the TEM images of the NGs and composite NGs with a 2% and 3% NZ content, respectively. Micrographs of the control NGs (PEGDA_700/2000_) shown in Figure 5a–d revealed agglomerates of NPs, with a spherical morphology, having dimensions roughly in the range 60–160 nm ± 10%, with an average size of about 115 nm. Micrographs of the composite NGs with 2% and 3% NZ content, shown in Figure 5e–l, indicate a morphology similar to that observed for the simple NGs, maintaining a spherical shape and having dimensions roughly in the range 100–200 nm ± 10%, with an average size of about 160 nm. This conclusion serves as confirmation of some of the dimensional details provided by the DLS technique.

**Figure 6:**
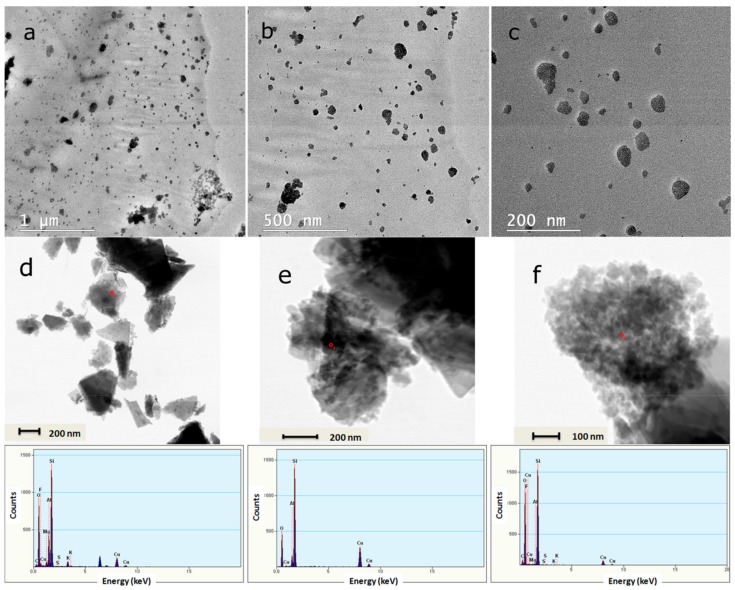
BFSTEM (**a**–**c**) and STEM (**d**–**f**) images for composite NGs after washing, lyophilization, and redispersion in distilled water and corresponding EDX analysis results of composite NGs.

The BFSTEM images shown in Figure 6 represent the composite NGs after washing the emulsion for the removal of emulsifiers, lyophilization, and redispersion in distilled water. The particle sizes are similar to those of particles appearing in the emulsion and maintain a spherical shape. Several points analysed by energy-dispersive X-ray analysis (EDX) highlighted the presence of the zeolite, through the appearance of peaks corresponding to its characteristic elements: Si, Al, Fe, etc.

#### 3.2.3. FTIR Measurements

**Figure 7:**
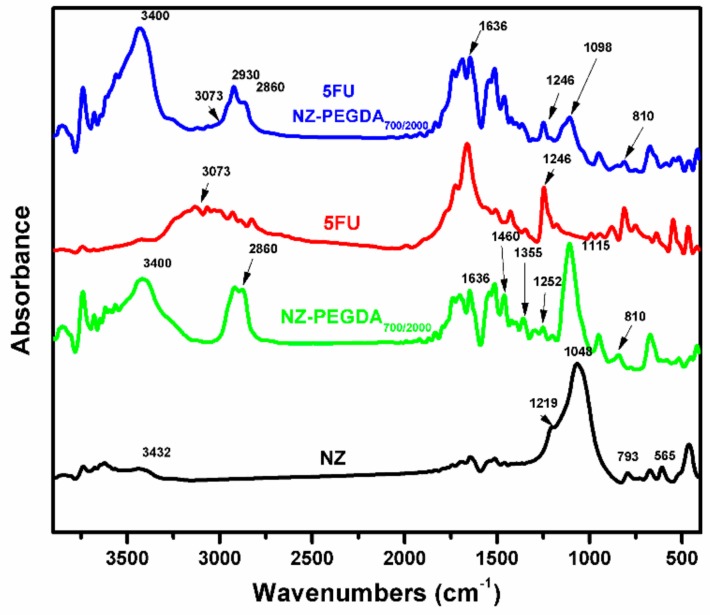
Fourier transform infrared FTIR spectra of zeolite (NZ), unloaded NG composite (NZ-PEGDA_700/2000_), 5-fluorouracil (5FU), and 5-fluorouracil-loaded NG composite (5FU 3NZ-PEGDA_700/2000_).

The FTIR spectra of simple zeolite (NZ), unloaded NG composite (NZ-PEGDA_700/2000_), 5-fluorouracil (5FU), and 5-fluorouracil-loaded NG composite (5FU NZ-PEGDA_700/2000_) are shown in Figure 7. The FTIR spectrum of simple NZ displayed the characteristic stretching vibrations of internal SiO_4_ tetrahedron units at 793 and 1048 cm^−1^ for zeolites. The broad band at around 3432 cm^−1^ is characteristic of the presence of H_2_O in the zeolite structure [37]. FTIR spectra of the composite NGs (NZ-PEGDA_700/2000_) revealed the characteristic bands of PEGDA displayed within the PEG backbone at 1115 cm^−1^, attributed to the C–O stretching vibrations [38]. The bands around 1255, 1355, 1460, and 2860 cm^−1^ are characteristic of the C–H stretching vibrations of CH_2_ and CH_3_ units, and those at 1636 cm^−1^ and at 810 cm^−1^ are characteristic of the asymmetric stretching of the acrylic bands –C=C, from the acrylate terminal group of PEGDA [39]. Regarding the composite NGs, characteristic bands of the zeolite overlapped with PEG bands, which led to an occultation [38]. FTIR spectra of the 5-fluorouracil-loaded composite NGs (5FU NZ-PEGDA_700/2000_) exhibited some of the characteristic bands of PEGDA and of the anticancer drug. The presence of 5-FU in the structure of the obtained nanocomposite was highlighted by the presence of particular bands at 3073 cm^−1^ (assigned to the stretching vibrations of the =C–H group) [40] and at 1246 cm^−1^ (assigned to the C–F bond vibrations) [24]. 

#### 3.2.4. TGA Investigation

**Figure 8:**
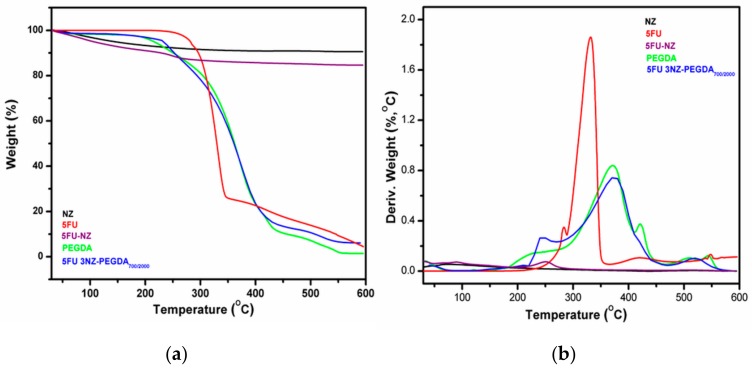
TGA (**a**) and DTG (**b**) curves of the zeolite (NZ), 5FU, PEGDA_700/2000_ macromer blend, 5FU-loaded zeolite particles (5FU-NZ) and 5FU-loaded NG composites (5FU 3NZ-PEGDA_700/2000_) samples.

The results of TGA and the corresponding DTG curves for simple zeolite (NZ), control NGs (PEGDA), 5-fluorouracil (5FU), and 5-fluorouracil-loaded composite NGs (5FU 3NZ-PEGDA) based on PEGDA_700_/_2000_ (3:1) macromer blend, are shown in Figure 8. The PEGDA nanogel denotes a complex decomposition process with a main event centred around 370 °C. The decomposition of PEGDA matrix ended at approximately 560 °C with no residue. The main degradation stage of the 5FU-loaded composite NZ-PEGDA_700/2000_ occurred at the same temperature as for the simple PEGDA hydrogel and corresponded to PEGDA degradation. The main difference between the 5FU-loaded composite NZ-PEGDA and the neat PEGDA hydrogel was the sharp degradation at around 240 °C, corresponding to 5FU decomposition. It can be noted that degradation of 5FU from the NZ–PEGDA composite occurred in the same temperature range as for the 5-FU from the loaded zeolite. This suggests that 5-FU in the hydrogel composite still interacts with the zeolite particles. The 4.5 wt% residue observed in the hydrogel composite corresponds to the zeolite content. Although the zeolite represented only 3% with respect to the macromer blend, the polymerization yield was below 100%, leading to a composite that is slightly richer in inorganic components.

#### 3.2.5. Swelling Behaviour

As summarized by Table 4, the swelling degree (SD) of control NGs and composite NGs obeys the same trend. The property increases as the molecular weight of used macromers increases. This is because shorter polymeric chains like those of PEGDA_700_ allow for a higher crosslinking degree, as a result of a greater number of available acrylate groups. Implicitly, a network with a higher crosslinking degree presents a lower swelling degree. Moreover, for NGs with NZ content, a decrease of the SD was observed. The NZ content is an important factor influencing the water absorption of the composite NGs, as part of the highly hydrophilic gel is replaced by a significant content of inorganic material with lower hydrophilicity and no swelling capability. However, the strong physical interaction between PEGDA and NZ particles that leads to the formation of new physical crosslinking points may also contribute to this decrease in the swelling capacity [41].

#### 3.2.6. Release Behaviour of the 5-FU-Loaded NG Composites

**Figure 9:**
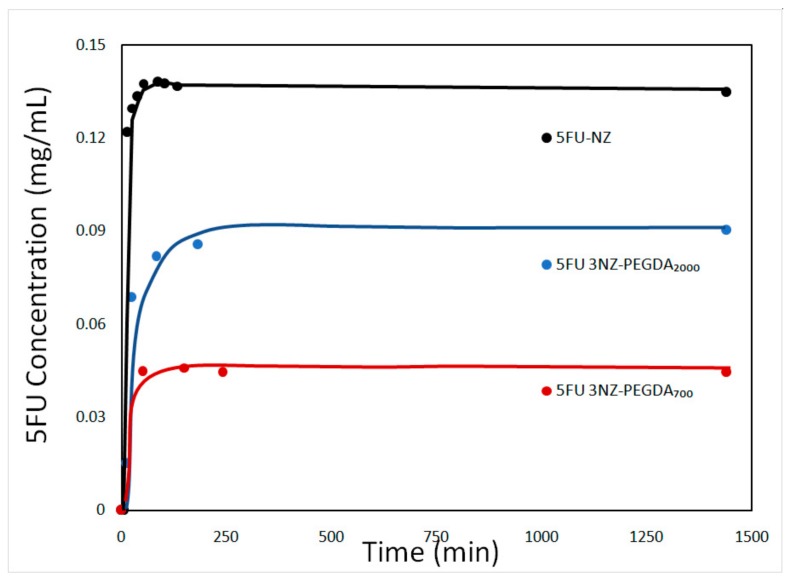
Release curves for 5FU-loaded zeolite and composite NGs with 3% NZ after 24 h.

The release profiles of NGs generally determine their role as nanocarriers for various biologically active formulations in anticancer therapies. Therefore, this study was mainly aimed at investigating the release rate of the encapsulated drug from the NG composites compared to the loaded NZs (Figure 9). 

As expected, the release of 5-FU from the NZ was faster compared to the NG composites. The release profile of 5-FU-loaded NZ showed burst release (of about 0.13 mg/mL) followed by a relatively slow release with an ascending slope up to ~4h, while the release profile of 5-FU-loaded NGs was similar to that of NZs, but with a lower burst (up to 0.08 mg/mL). The overall release process of NGs was dominated at first by the diffusion of 5FU from the zeolite’s pores into the NG network, followed by diffusion from the crosslinked network into the simulated biological medium. After 24h, the maximum quantity of drug released plateaued at around 40–45%with respect to the loaded 5FU for all the studied NG composites. We hypothesize that the release was not complete due to an equilibrium being reached between the buffer system and the NGs matrix. Studies concerning NGs employed in cancer treatment indicate that avoiding opsonization and clearance by the MPS system by coating of an inert compound, such as PEG [42,43], allows nanogels to have extended circulation half lives [44]. This gives nanogels enough time to accumulate at the tumour site and begin releasing their load. Although cellular uptake studies are needed to make definitive conclusions, the inert coating and the size of the composites are early indicators of their potential use as extended 5FU release vehicles.

In composite NGs, the investigated parameter of significant influence was the MW of PEGDA chains. In agreement with the strong drug–network interactions, the drug release rate was smaller for shorter PEGDA chain hydrogels, meaning PEGDA_700_. The effect of the network mesh size can also slow down the release of the drug with decreasing PEGDA molecular weight [45,46]. Hence, the drug was released more slowly from the NG with the higher cross-linking degree (i.e., NZ-PEGDA_700_), as shown in Figure 9. The results revealed that NG composites are endowed with a high loading capacity of 5-FU and slow delivery of drug, which is an important feature in treating most types of cancer. 

Analysing the release data by means of the four mathematical models described in the experimental part, this study aims at gaining some insight into the kinetics of drug release from NZ particles and NG composites. The fitting was evaluated through the coefficient of determination (*R^2^*) computed for each sample and for each fitting model, as summarized in Table 5. For all the studied cases, the Korsmeyer–Peppasmodel was the most suitable mathematical model to describe the release. This is quite reasonable as the model corresponds to the kinetic description of low-molecular-weight compounds diffusing from porous matrices. The Higuchi model also describes the kinetic of low-molecular-weight compounds diffusing from porous matrices, yet the fitting was lower. This is due to the fact that the Higuchi does not account for any dissolution of the polymeric matrix and only allows for minor changes in the shape and size of the system. By contrast, the Korsmeyer–Peppas model considers both the dissolution events, as well as the swelling behaviour that is characteristic of the proposed NG composites. The only explanation for the slight deviation of the experimental data from the theoretical model is that the Korsmeyer–Peppasmodel assumes a cylindrical shape of the “tablet” from which the drug is released, while the proposed NG composites show spherical morphologies. Although another work [25] proposes the 0.5–0.9 interval for the coefficient corresponding to Fickian diffusion, *n*, from spherical particles (close to the ones calculated for 5FU NZ-PEGDA_700_ and 5FU NZ-PEGDA_2000_ samples), its values can decrease down to 0.2–0.3 for polydisperse systems [47], as obtained for the 5FU-NZ and 5-FU NZ-PEGDA_700/2000_ samples.

#### 3.2.7. Cytotoxicity Evaluation of the 5FU-Loaded NG Composites

The cytotoxicity of unloaded nanogels was compared to that of the loaded composites in equivalent concentrations, as well as the free drug formulations. A one-factor analysis of variance (ANOVA) was performed, which is usually recommended when running multiple tests, and revealed that there is a statistical significance between the means of the studied groups (*p* < 0.1%). However, Student’s *t*-test was preferred to compare relevant groups and correct the statistical significance to account for the multiple tests being run. 

**Figure 10:**
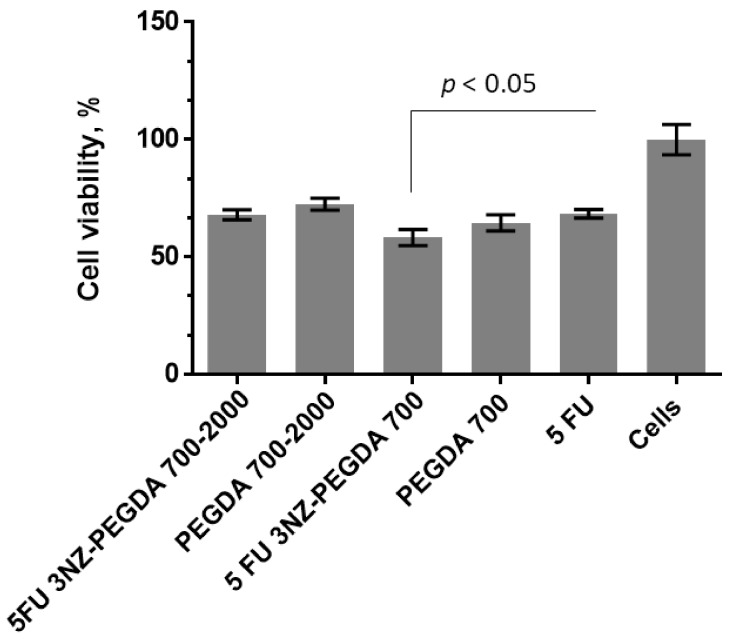
Cell viability variation of the L929 cell line incubated in the presence of unloaded PEGDA_700/2000_ nanogels, PEGDA_700_ nanogels, 5 FU 3NZ PEGDA_700/2000_ nanogels and 5FU 3NZ-PEGDA_2000_ nanogels and free 5 FU in an equivalent concentration.

Comparing the cell viability of the samples incubated with the unloaded composite and the control NGs, a decrease was observed for NG composites (Figure 10), particularly for the NG composites prepared with PEGDA_700/2000_. However, because the dilution was considerable, the differences were rather moderate, and did not appear to be statistically significant. The behaviour of composite and control NGs was rather similar when PEGDA_700_ was used for preparing the NGs. Statistically significant differences were observed between the PEGDA_700_-loaded composite and the free drug formulation in equivalent concentrations. Although the NGs developed in this study are not ideal in terms of PDI (meaning they are not monodisperse, PDI=0), the cytotoxicity tests performed in duplicate for a fixed dose of NGs have indicated good reproducibility concerning the cell viability, particularly for 3NZ-PEGDA_700/2000_ with the lowest PDI value (0.180), according to Table 3. Hence, in this case, the PDI of NGs did not have a significant influence on the cell viability. However, the effect may differ as different doses of NGs are applied. A direct effect of NGs’ polydispersity on cell viability is difficult to quantify, as the concentration of NGs also brings about significant changes in cell viability.

**Figure 11:**
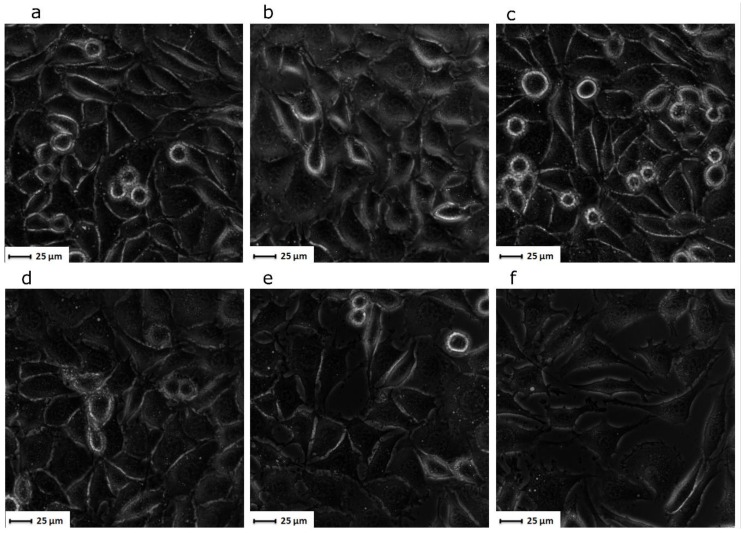
Representative optical microscopy images of L929 (**a**); L929 cell line exposed to unloaded control NGs (**b**) with PEGDA_700_, (**c**) with PEGDA_700/2000_; L929 cell line exposed to 5FU-loaded composite NGs (**e**) with NZ-PEGDA_700_, (**f**) with NZ-PEGDA_700/2000_; and L929 cell line exposed to concentrated 5FU solution (**d**).

Furthermore, when analysing the cell viability exposed to loaded NGs (Figure 11) against an equivalent concentration of free drug, the cytotoxicity seemed to be enhanced by the encapsulation of the drug. As observed from the optical microscopy (Figure 11), the cells exposed to the control NGs present minor morphological differences compared to the cells alone. However, the cells contacted with loaded NG composites (particularly with PEGDA_700/2000_) presented a related morphology to that of cells in direct contact with the concentrated solution of 5-FU. Hence, for this cell line, there are indicators showing that loaded NGs produce an increased cytotoxic effect. In addition to colorimetric cell viability analysis, the effect of 5-FU-loaded NGs composites was also observed via optical microscopy. When comparing the cells exposed to loaded NGs with cells exposed to an equivalent concentration of free drug, the cytotoxic effects seem to be enhanced by the encapsulation of the drug (Figure 11). Cells exposed to control NGs present few to no differences in terms of morphology and density compared to untreated cells. However, L929 cells that were treated with loaded NG composites (particularly in the case of PEGDA_700/2000_ NGs) were more loosely packed and less confluent compared to all other conditions, including direct contact with a concentrated solution of 5-FU. Hence, for this cell line, there are indicators showing that loaded NGs produce an increased cytotoxic or antiproliferative effect. More studies looking into the cellular uptake of nanogels and their potential pathways have to be performed to determine whether the administration of the drug in an encapsulation form enhances the local cytotoxicity. This can only be explained by the antiproliferative character of the drug, which acts not by inducing the apoptosis of cells but by stopping their proliferation cycle.

Overall, the results are encouraging; in addition to the cytotoxic/antiproliferative effect observed in Figure 10 and Figure 11, the NG composites have potential as drug carrier systems, particularly in stroma cells of solid tumours. However, due to the observed effects of the unloaded gels, more work has to be dedicated to a better purification method, to ensure the carriers themselves are biocompatible and do not affect normal cells. Consequently, this system might be promising to be investigated in cancer cell lines as well.

## 4. Conclusions 

The study focused on developing new composite NGs based on cross-linked PEGDA and NZ loaded with 5FU, as a bioactive substance used in cancer treatment. The NGs were obtained by polymerization in an inverse mini-emulsion using PEGDA_2000_, PEGDA_700_, or a blend of the two (in a 1:3 ratio), in the presence of different NZ ratios. The system has been shown to be prone to Ostwald ripening and was controlled by adding NaCl as a co-stabilizer. DLS measurements confirmed polymerization in the nanodroplets, dispersed in the organic continuous medium, characteristic of(inverse) mini-emulsions. With an up to 3% content of NZ, the composites remained in the range of 100–200 nm average diameters, being well suited for the envisioned application. The presence of NZ in the composite NGs was confirmed and quantified by EDX and TGA, respectively. The release profile of 5-FU-loaded NG composites and 5-FU-loaded NZ was also studied. Composites presented slower release rates of the drug, with a lower burst at the beginning of the study. Of the four mathematical models proposed for the kinetics of release, the Korsmeyer–Peppas model fitted all the NGs and the loaded NZs best; the dominant release mechanism of drugs has been proven to be Fickian diffusion. Cytotoxicity tests on the L929 cell line of murine fibroblasts indicated a reduction in cell viability to a value of 60% for the NG composites diluted at a ratio of 1/10. This value was slightly lower compared to a 5-FU solution of equivalent concentration, yet the results indicated the potential for maintaining a similar cytotoxic effect in an encapsulated form of NZ-based NGs. Based on the overall results, the NZ-based NGs can serve as potential candidates for controlled drug delivery.

## Figures and Tables

**Table 1:** The synthesis recipes for control NGs and composite NGs.

Sample	PEGDA_700_/PEGDA_2000_	Zeolite (wt%)
**PEGDA_700_**	100/0	**0**
**PEGDA_2000_**	0/100	**0**
**PEGDA_700/2000_**	75/25	**0**
**1NZ-PEGDA_700_**	100/0	**1**
**1NZ-PEGDA_2000_**	0/100	**1**
**1NZ-PEGDA_700/2000_**	75/25	**1**
**2NZ-PEGDA_700_**	100/0	**2**
**2NZ-PEGDA_2000_**	0/100	**2**
**2NZ-PEGDA_700/2000_**	75/25	**2**
**2NZ-PEGDA_700_**	100/0	**3**
**2NZ-PEGDA_2000_**	0/100	**3**
**3NZ-PEGDA_700/2000_**	75/25	**3**

**Table 2:** Mean particle size of NG composite particles measured by DLS and conversion.

Sample	Lipophobic Agent	SPAN 80 Concentration (wt%)	Mean Particle Size (nm)	Conversion(%)
PEGDA_700_	-	-	195 ± 8.1	15%
PEGDA_700_	NaCl	-	197 ± 19.9	89%
PEGDA_700_ *	NaCl	[SPAN 80] = 5%	179± 5.9	78%
PEGDA_700_ **	**NaCl**	[SPAN 80] = 5%	**189 ± 19.3**	**99%**
PEGDA_700/2000_	NaCl	[SPAN 80] = 5%	154± 8.6	69%
PEGDA_2000_	NaCl	[SPAN 80] = 5%	178 ± 13.8	46%
3NZ-PEGDA_700_	-	-	171 ± 69	10%
3NZ-PEGDA_700_	NaCl	[SPAN 80] = 5%	19± 24.6	85%
3NZ-PEGDA_2000_	NaCl	[SPAN 80] = 5%	17± 10.2	37%
**3NZ-PEGDA_700/2000_**	**NaCl**	[SPAN 80] = 5%	**197 ± 14.2**	**91%**

* after 24 h reaction time; ** after 42 h reaction time.

**Table 3:** DLS results of composite NGs with varying percentages of encapsulated NZ particles.

Sample	NZ (wt.%)	Mean Particle Size (nm)	PDI
NZ	100	148 ± 7.7	0.577
1NZ-PEGDA_700_	1	203 ± 0.5	0.187
1NZ-PEGDA_2000_	1	166 ± 1.1	0.189
1NZ-PEGDA_700/2000_	1	153 ± 0.5	0.183
2NZ-PEGDA_700_	2	195 ± 0.7	0.200
2NZ-PEGDA_2000_	2	225 ± 9.2	0.267
2NZ-PEGDA_700/2000_	2	189 ± 1	0.173
3NZ-PEGDA_700/2000_	3	192 ± 0.6	0.180

**Table 4:** Swelling degree (SD) of studied samples.

	Sample	SD (%)
**Nanogels**	PEGDA_700_	210± 0.5
	PEGDA_700_/_2000_	296± 0.2
	PEGDA_2000_	439± 1
**Composite Nanogels**	3NZ-PEGDA_700_	160± 0.5
	3NZ-PEGDA_700/2000_	257± 1.5
	3NZ-PEGDA_2000_	388± 0.8

**Table 5:** Fitted parameters of the kinetic models used in the 5-FU slowrelease of sample.

Mathematical Models	5FU-NZ	5FU NZ-PEGDA_700_	5FU NZ-PEGDA_700/2000_	5FU NZ-PEGDA_2000_
**First-order**				
K min^−1^	0.00018	0.00011	0.00015	0.00016
*R^2^*	0.6810	0.5672	0.7309	0.5738
**Zero-order**				
K_0_, µg/mL	0.0196	0.0196	0.02	0.0238
*R^2^*	0.927	0.788	0.731	0.773
**Higuchi**				
K_H_, µg/mL∙min^−1/2^	2.055	2.136	1.212	2.650
*R^2^*	0.982	0.899	0.976	0.914
**Korsmeyer–Peppas**				
K	3.306	2.321	6.087	1.736
*n*	0.319	0.406	0.256	0.450
*R^2^*	0.988	0.963	0.984	0.968
